# Micromilling vs hand drilling in stable isotope analyses of incremental carbonates: The potential for δ^13^C contamination by embedding resin

**DOI:** 10.1002/rcm.9318

**Published:** 2022-05-23

**Authors:** Tansy Branscombe, Julia Lee‐Thorp, Rick Schulting, Melanie Leng

**Affiliations:** ^1^ School of Archaeology University of Oxford Oxford UK; ^2^ National Environmental Isotope Facility British Geological Survey Nottingham UK; ^3^ School of Biosciences University of Nottingham Loughborough UK

## Abstract

**Rationale:**

Embedding resins are commonly used to facilitate high‐resolution sampling for stable isotope analysis but anomalous δ^13^C values have been observed in some cases. Here we compare the results of microsampling strategies for hand‐drilled versus resin‐embedded micromilled samples from the same marine shells to assess whether resin contamination is implicated in δ^13^C spikes. The comparison allows assessment of the relative benefits for spatial resolution, seasonal range for both δ^18^O and δ^13^C, and sample failure rates.

**Methods:**

Hand‐drilled samples were obtained from two bivalve shells (*Spisula sachalinensis*), corresponding to micromilled samples on the same shells where high δ^13^C spikes were observed. All carbonate powders were analysed using a dual‐inlet Isoprime mass spectrometer and Multiprep device. Results from both sample sets were compared statistically.

**Results:**

No anomalous high δ^13^C values and no failures due to insufficient gas were observed in the hand‐drilled samples in contrast to the embedded micromilled sequences. Spatial resolution was reduced (~2.5×) in the former compared with the latter, resulting in a small reduction in the total range observed in the micromilled δ^13^C and δ^18^O values. Reduced sampling resolution between the two datasets was only significant for δ^18^O.

**Conclusions:**

For *S. sachalinensis* (as with other similar bivalves), rapid growth mitigates the reduced sampling resolution of hand drilling and does not significantly impact observed isotopic range and seasonal patterning. Occurrence of anomalous δ^13^C values were eliminated and failure rates due to insufficient sample size greatly reduced in the hand‐drilled dataset. We can find no other explanation for the occurrence of δ^13^C spikes than contamination by the embedding agent. We conclude that the logistical and interpretational benefits of careful hand drilling may be preferable to resin embedding for micromilling in marine shells, corals or speleothems where growth rate is rapid and the highest resolution is not required.

## 1 INTRODUCTION

Carbon and oxygen stable isotope (δ^13^C/δ^18^O) analysis of marine bivalves can be used to provide (palaeo)environmental information on marine conditions, including water temperature,^1^, ^2^, ^3^, ^4^ salinity^5^ and productivity,^6^, ^7^ as well as biogeographic information on the species analysed.^8^, ^9^, ^10^ Depending on the size and shape of the material/species being sampled, and the resolution and precision requirements of the study, a variety of sampling techniques can be used to produce powder samples for mass spectrometry analysis. These have been discussed by other authors, primarily referring to hand drilling (sometimes known as microdrilling, but referred to as hand drilling throughout this paper), micromilling, SIMS (Secondary Ion Mass Spectrometry) and laser ablation techniques in the sampling of marine and freshwater carbonates such as mollusc shells, corals and otoliths,^9^, ^11^ as well as in the related field of speleothem research.^12^, ^13^, ^14^, ^15^

Although SIMS and laser ablation are increasingly used, hand drilling and micromilling remain the most commonly used sampling methods,^11^, ^16^ due to their balance of accessibility, precision and resolution, and because they produce the powdered samples necessary for high‐precision MC‐ICPMS (Multicollector Inductively Coupled Plasma Mass Spectrometry) and ICP‐AES (Inductively Coupled Plasma Atomic Emission Spectroscopy).^17^, ^18^, ^19^ Micromilling provides the opportunity to precisely mill from very specific regions of the sample and at high resolution, but is more time consuming than hand drilling and the equipment is often temperamental. The collection of micromilled powder samples is most commonly accomplished manually using a combination of scalpels and razor blades,^16^ so the process requires a flat and relatively wide sampling surface. This means that in order to be micromilled, most materials need sectioning in order to provide such a surface. In the case of fragile or small/thin material (including, e.g., shells and otoliths) this also necessitates embedding specimens in a stabilising medium to make them less susceptible to breakage, and to increase the size of the flat surface available when collecting samples. The embedding and sectioning process can be laborious, further increasing the time and resources required for this approach.

By contrast, hand drilling is expedient and inexpensive. Depending on the size of the drill bit, it usually produces larger sample sizes, although with lower resolution due to increased time averaging within and between samples. Unlike micromilling, hand drilling can be performed on flat or curved surfaces. This allows sampling to be done around the curve of a shell and reduces the need for extensive preparation of the sampling surface.

This study was prompted by the results of δ^13^C and δ^18^O analysis on archaeological samples of the marine bivalve *Spisula sachalinensis* – a large and long‐lived bivalve found in the northwest Pacific.^20^ Results were obtained from a resin‐embedded specimen which had been sampled using a computer‐operated New Wave Research MicroMill. δ^18^O values show a seasonal annual temperature curve as expected, but the δ^13^C record of two of the seven shells showed regions where the values were significantly more positive than expected (up to ~5‰). These positive spikes were especially apparent when compared with the rest of the sequences from these individual shells, as well as results from other shells analysed, where δ^13^C is relatively stable at ~ +1 ± 0.5‰.

*S. sachalinensis* is a well‐researched species,^20^, ^21^, ^22^, ^23^, ^24^, ^25^, ^26^, ^27^, ^28^, ^29^ and sudden positive δ^13^C excursions of this nature are ecologically implausible. The species lives above the thermocline at depths <15 m,^23^, ^28^, ^29^, ^30^ where there is not a high degree of seasonal change in surrounding seawater DIC (dissolved inorganic carbon). We therefore expect relative stability in shell δ^13^C values. Moreover, δ^13^C values as high as ~5‰ would be considered implausibly high in all cases for marine bivalves. In McConnaughey and Gillikin's^6^ overview of the physiological and environmental controls affecting shell δ^13^C values, there is no mention of any condition or species where marine shell δ^13^C would near 5‰, nor any mechanism that could account for such large and sudden spikes in δ^13^C. We are unaware of any literature that reports shell δ^13^C values this high and considers it a true reflection of the growing shell's isotopic composition. The large δ^13^C excursions seen in these two specific shells (labelled II_1 and VIII_3) discussed in this paper were therefore considered highly anomalous, leading us to investigate the potential role of sample preparation and sampling strategy in affecting δ^13^C results.

As the micromilled shells were embedded in polyester resin prior to sampling, one possible consideration is resin contamination. Polyester and epoxy resins are both complex organic polymers^31^ commonly used in stable isotope analysis of carbonates to impregnate or embed materials and enable sampling. Despite the widespread use of these resins, and the fact that the tiny carbonate sample sizes mean that even miniscule contaminant contributions could significantly influence isotope results, research into their potential as an isotopic contaminant is uncommon. Mortensen et al^32^ undertook a direct comparison between δ^13^C and δ^18^O values in aragonite coral septa, analysing a series of resin‐embedded septa alongside a non‐embedded control sample. They saw a 0.12‰ enrichment in δ^18^O and a 0.17‰ depletion in δ^13^C, but in the context of their analysis this was not considered significant as these differences were smaller than their inter‐sample variation. This study has since been cited as evidence that polyester resin ‘does not contribute to the CO_2_ evolved by acid digestion that is used for the isotopic measurements’.^33^

However, the study of Mortensen et al^32^ dates to 1998, and the effects of a 0.12‰ δ^18^O enrichment and a 0.17‰ δ^13^C depletion are greater than the average analytical precision of modern mass spectrometry, which is usually ~ ±0.06‰ for carbonate samples (e.g. Radke et al^34^). At the British Geological Survey (BGS), where the isotopic analysis used in this study was carried out, precision is routinely <0.05‰. A more recent study by Schöne et al^35^ investigated a number of different pretreatment methods and sample preparation materials, finding that contamination of carbonate standards by commonly used resin ‘glues’, including the Kleer‐Set resin discussed in this paper, can produce *at least* moderate isotopic shifts. Furthermore, Rodríguez de Vera et al^31^ suggest that polyester resins can have a significant ‘interference effect’ on compound‐specific mass spectrometry of lipids in archaeological sediments, particular affecting δ^13^C_18:0_. Resin‐associated analytical issues have also been previously noted by the BGS in high‐resolution speleothem work, but these were never fully investigated. This existing evidence highlights the issue of resin contamination in carbonate palaeothermometry, but has not yet dissuaded the common use of sample pretreatment, embedding, and gluing in subsequent studies (e.g. ^36^, ^37^). Moreover, previous studies do not explore whether changes to sampling strategy to avoid such contaminants results in an unacceptable loss of precision and/or resolution.

Here we tested corresponding hand‐drilled, non‐embedded samples from the same valves to assess whether the high δ^13^C values occurred in both datasets. If so, then we must re‐examine our initial position and examine how these high δ^13^C results could be otherwise explained. Alternatively, if these δ^13^C peaks are not present in the hand‐drilled samples then we can consider how sampling strategy and/or polyester resin contamination are potential issues for future carbonate stable isotope analyses. This would have relevance not only to the shells used in this particular study, but also to any researcher contemplating the use of embedding resin in a strategy for high‐resolution sampling of carbonates for stable isotopes.

## 2 METHODS

**FIGURE 1:**
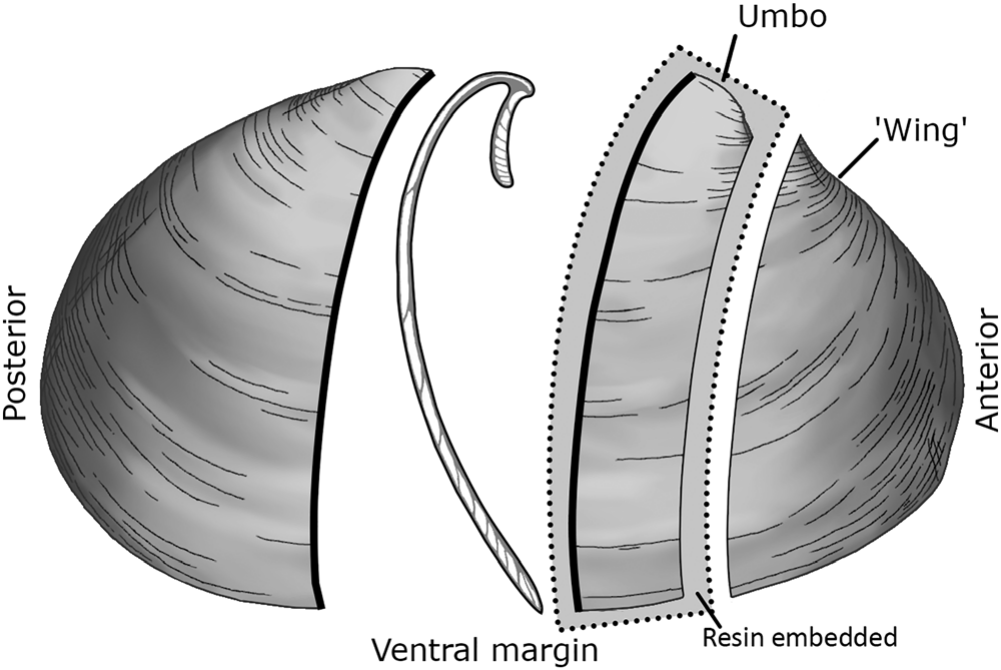
Illustration of *Spisula sachalinensis* showing section along the axis of maximum growth. The bolded lines show the sampled surfaces

The samples used in this experiment came from two archaeological *S. sachalinensis* valves (labelled VIII_3 and II_1) both collected from the site of Hamanaka 2, Rebun Island, Japan. In the initial stable isotope analysis undertaken on these shells, the valves had been sectioned as per Figure 1, with the central section of the shell (corresponding to the axis of maximum growth) then embedded in MetPrep ‘Kleer‐Set’ polyester resin (https://metprep.co.uk/product/kleer-set/). Samples were milled incrementally from these resin‐embedded sections using a computer‐controlled New Wave Research MicroMill at the BGS. Milling was carried out up to 300 μm depth in multiple milling passes of 50 μm depth/pass, and using a 0.3 mm diameter diamond‐coated dental drill bit. No chemical or physical pretreatments were applied to the samples before analysis. The non‐embedded halves of the same valves were sampled using a Buehler hand drill, from the area corresponding to the original sampling locations (Figure 3). For both the micromilled and hand‐drilled samples, the cross‐lamellar layer of the shell was targeted, as is most common with shell palaeothermometry studies.^38^ In this large species, the cross‐lamellar layer is relatively thick (ca. 4 mm width after the second year of growth; Figure 3), so it was possible to avoid mixing material from the surrounding outer and inner layers by eye during hand drilling. In other smaller species of shell, it may be more difficult to specifically target one microstructural layer during hand drilling. Samples were spot drilled using a 1 mm diameter drill bit, and aluminium foil was used to collect the resulting aragonite powder before transferring it into microcentrifuge tubes. During hand drilling, the shell was supported using the non‐dominant hand, which rested firmly on the lab bench and provided a ‘cushion’ between the shell itself and the bench to prevent direct contact between the two which could cause damage to the specimen. The sectioned surface of the shell was held angled down towards the aluminium foil to direct the shell powder onto the foil and reduce sample loss. Each sample was checked for purity of the aragonite using Fourier‐transform infrared spectroscopy,^20^ as per Loftus et al.^39^

Once in the microcentrifuge tubes, the powders obtained using both the hand‐drilling and micromilling methods were analysed identically, using a dual‐inlet Isoprime mass spectrometer interfaced with a Multiprep autosampler (hereafter referred to as the Isoprime plus Multiprep) at the BGS. We aimed to produce 50 to 100 μg of carbonate per sample for isotope analysis; however, micromilled samples were often below 50 μg, smaller than those produced by hand drilling which were typically >100 μg (with 50–100 μg sub‐samples used for analysis). Samples are loaded into glass vials and sealed with septa, evacuated and anhydrous phosphoric acid delivered to the carbonate at 90°C. Evolved CO_2_ is collected for 15 min, H_2_O is removed, and pure, dry CO_2_ introduced into the mass spectrometer for measurement. Isotope ratios of carbon and oxygen (^13^C/^12^C, ^18^O/^16^O) are expressed as per mille (‰) deviations of the isotopic ratios (^13^C/^12^C, ^18^O/^16^O) calculated to the VPDB (Vienna Pee Dee Belemnite) scale using a within‐run laboratory standard (KCM) calibrated against international standard NBS‐19. Nine KCM calcite standards were measured alongside every 31 samples. The Craig correction^40^ was applied to correct for δ^17^O, as well as a calcite‐acid fractionation factor of 1.00813. Due to the long run time of 21 h a drift correction is applied across the run, calculated using the standards that bracket the samples. The standard calcite values for KCM are +2.00‰ for δ^13^C and −1.73‰ for δ^18^O with an average analytical reproducibility of 0.05‰.

A small aliquot of the resin was analysed separately on an Elementar vario ISOTOPE cube elemental analyser (EA) coupled to an Isoprime precisION isotope ratio mass spectrometer also at the BGS, in an attempt to provide a baseline δ^13^C isotope ratio for the embedding resin. The significant drawback to this approach is that the resin is fully combusted, including organic components, while the phosphoric acid in the Multiprep preferentially reacts with carbonates, and therefore likely does not dissolve all components. Therefore, this was not the best approach to determine the effects of resin traces on the shell carbonates; it would have been preferable to analyse the resin sample on the Multiprep, but this ran the risk of a costly contamination of the instrument and could not be justified.

## 3 RESULTS AND DISCUSSION

**FIGURE 2:**
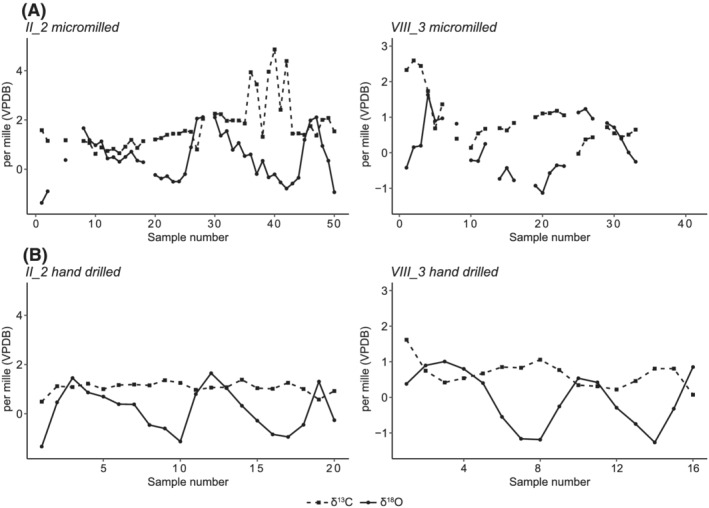
Plots showing the δ^13^C and δ^18^O results for shells II_1 and VIII_3 for A, the micromilled samples and B, the hand‐drilled samples

Of the 36 hand‐drilled samples added for this paper, all produced sufficient gas for isotopic measurement. Of the pre‐existing micromilled samples from these two shells, 21 of the 91 samples failed to produce enough gas to measure isotopic composition. This represents a failure rate of 23%, likely due to challenges with handling very small amounts of powder during the period between micromilling and mass spectrometry. The results of all (hand‐drilled and micromilled) samples are summarised in Table 1. The δ^18^O results show clear cyclicity, with a range of 2.98‰ for shell II_1 and 2.28‰ for VIII_3. The δ^13^C results for hand‐drilled samples show a smaller range of 0.87‰ for II_1 and 1.55‰ for VIII_3. Figure 2 shows the results of the hand‐drilled samples alongside the original results from the corresponding micromilled samples.

**TABLE 1:** Summary of stable isotope results from shells II_1 and VIII_3

		Maximum δ^18^O (‰)	Minimum δ^18^O (‰)	δ^18^O range (‰)	Maximum δ^13^C (‰)	Minimum δ^13^C (‰)	δ^13^C range (‰)
II_1	Micromilled/resin‐embedded	+2.11	−1.37	+3.48	+4.86	+0.62	+4.24
	Hand drilled/unembedded	+1.65	−1.33	+2.98	+1.36	+0.49	+0.87
VIII_3	Micromilled/resin‐embedded	+1.63	−1.13	+2.76	+2.59	−0.03	+2.62
	Hand drilled/unembedded	+1.01	−1.27	+2.28	+1.62	+0.07	+1.55

**FIGURE 3:**
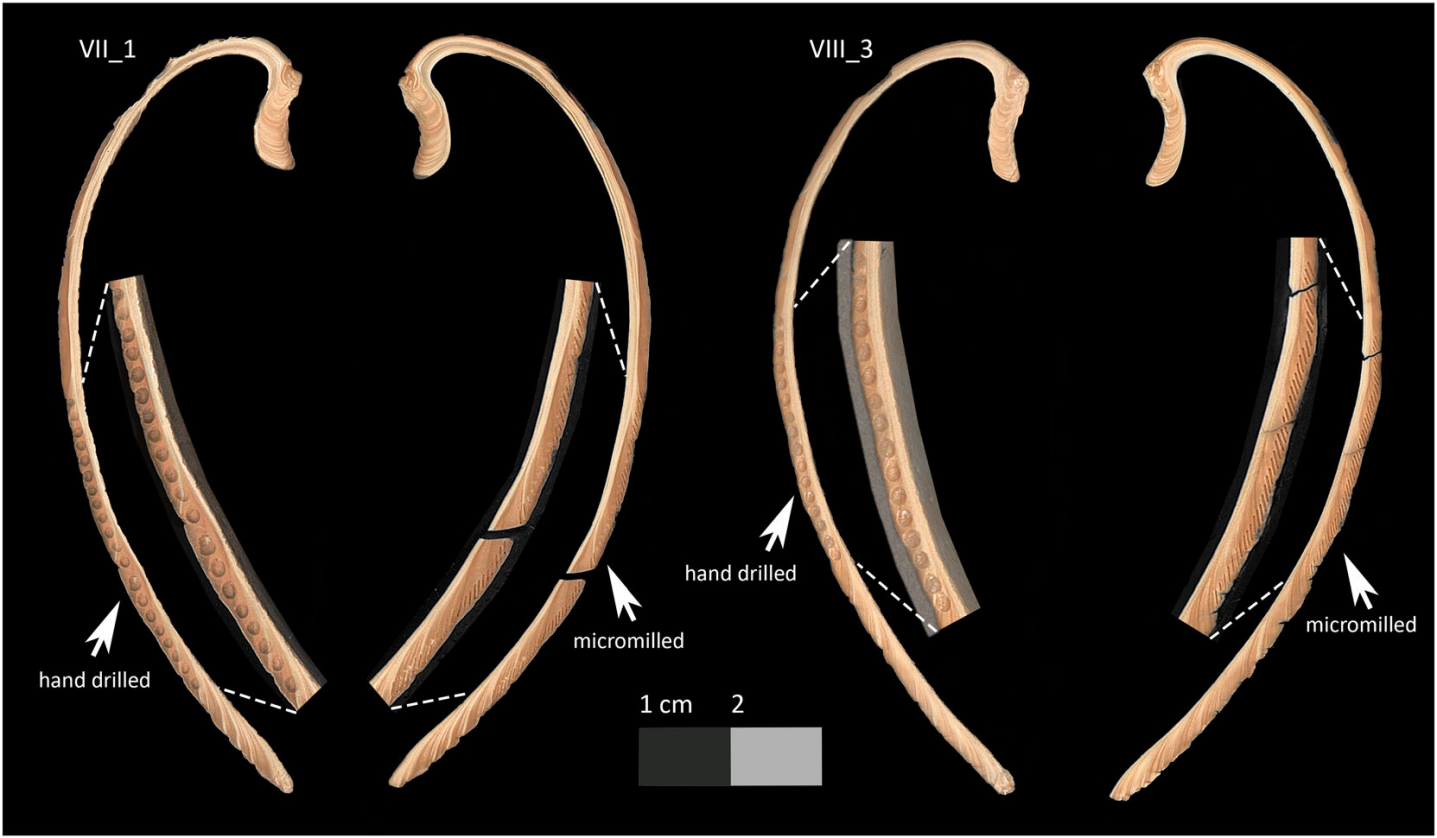
Scan of sampled sections for shells II_1 and VIII_3 showing the corresponding micromilled and hand‐drilled regions [Color figure can be viewed at wileyonlinelibrary.com]

Compared with the micromilled samples, the hand‐drilling approach provided ~2.5× lower spatial sampling resolution. Shell II_1 produced 20 hand‐drilled samples over the same area that produced 50 micromilled samples, and VIII_3 produced 16 hand‐drilled samples compared with 41 micromilled. The difference in resolution relates to the relative size of the drill bits used in each case (0.3 mm for micromilling vs 1 mm for hand drilling), and the increased spatial precision obtainable when using a computer‐operated system. These results, unsurprisingly, show that hand drilling results in reduced sampling resolution compared with micromilling, which is one of the main reasons cited by Twaddle et al^11^ that micromilling is the method of choice for most researchers. In this study, hand drilling produced a resolution of approximately one sample per 1.7 mm distance along the shell. Drill bits smaller than 1 mm diameter could be sourced to increase the resolution of hand drilling, but the spatial precision achievable by hand drilling will still be limited by the steadiness and coordination of the drill operator, and the fragility of increasingly small drill bits. Furthermore, this study does not represent the maximum spatial precision achievable with micromilling, as we sampled using discrete consecutive milling trenches (as seen in Figure 3) with some material remaining between each sample. Other researchers have shown that continuous sampling can be achieved where each sampling pass is less wide than the diameter of the drill bit, no material remains between samples, and spatial resolution can go under 100 μm.^12^, ^14^

**FIGURE 4:**
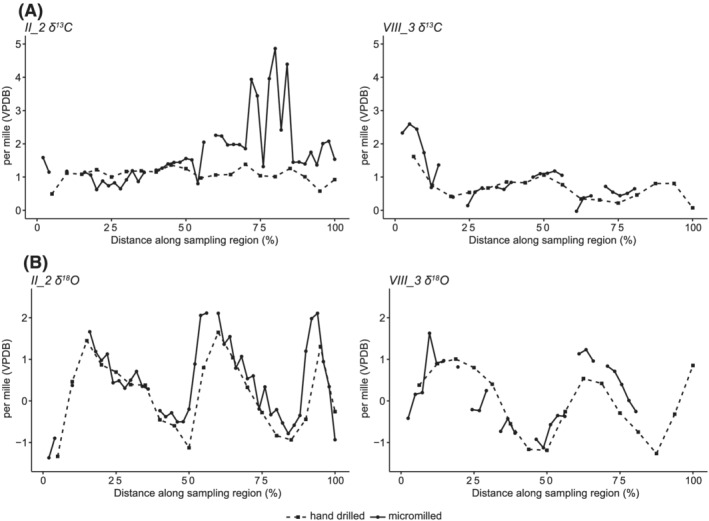
Plot showing A, δ^13^C results from hand‐drilled and micromilled samples plotted against distance along the shell (as a percentage along the sampling region) and B, δ^18^O results from hand‐drilled and micromilled samples plotted against distance along the shell

Figure 4 shows a close fit between the δ^18^O data for both sequences, with the aforementioned slightly reduced δ^18^O ranges seen in the hand‐drilled sequences for both shells (Table 1). For shell II_1 the δ^18^O range for the non‐embedded hand‐drilled samples was reduced by 0.50‰, and for VIII_3 the range was similarly reduced by 0.48‰. This means that for II_1 and VIII_3, respectively, the δ^18^O ranges of the hand‐drilled samples were 85.6% and 82.6% of the micromilled samples. A reduction of 0.5‰ in δ^18^O equates to a reduction of ~2°C in the calculated temperature range, using the aragonite/temperature equation from Grossman and Ku,^3^ as modified by Leng and Lewis.^41^ Therefore, if the intended research outcome requires very precise calculations of maximum and minimum ocean temperatures then hand drilling is likely to dampen these extremes (especially in slow‐growing species) and micromilling is therefore preferable. However, if the intended outcome is to identify seasonal cycles or season of collection then higher resolution may be surplus to requirements.

Despite the lower spatial resolution and compression of δ^18^O values in the hand‐drilled samples, the sequences appear more coherent and are easier to interpret in terms of seasonal shifts compared with the micromilled results (Figure 2). This is at least partly because the increased resolution achieved in the micromilled results (Figure 2A) is more complex and appears ‘noisy’, rendering seasonal ‘patterns’ more difficult to interpret as compared with the hand‐drilled results (Figure 2B). The gaps in the micromilled results further contribute to this difficulty, whereas the hand‐drilled sequence is continuous. We believe the problem relates to the smaller sample sizes obtained via micromilling, and subsequent powder loss when transferring the powder between vessels, so that failure rate due to small quantities of CO_2_ is relatively high. Hand‐drilled samples produced material easily in or above the 50–100 μg range typically run on the Isoprime plus Multiprep device, while micromilling regularly produced smaller samples of ~30 μg (weighed in microcentrifuge tubes immediately after sample collection). Small samples such as this are harder to transfer and powder will be lost due to static on transfer between microcentrifuge tubes and septa. Larger samples have a much lower failure rate, and in the case that samples do fail there is usually enough leftover powder to repeat those samples. The sample sizes obtained through micromilling could be increased by targeting larger areas or drilling deeper into the shell section, but this would compromise the sampling precision (one of the main benefits of micromilling), while prolonging the already‐slow milling time. Similarly, statistically smoothing the data (e.g. ^42^) to reduce the apparent noise obviates the point of having higher sampling resolution.

The differences between the micromilled and hand‐drilled samples were greater for the δ^13^C results. The range of δ^13^C for the hand‐drilled samples was reduced by 3.37‰ for II_I and by 1.07‰ for VIII_3. In both cases, the difference is due to a reduction in the maximum observed δ^13^C value. This strongly suggests that the highest δ^13^C values observed in the original micromilled samples are indeed erroneous. As they were not replicated in the hand‐drilled results it suggests that this issue was resolved by the change in sampling strategy to hand drilling unembedded material. The other possibility, that the high δ^13^C values were completely missed in the hand‐drilled sampling spots, is very remote. This is even more the case since the sampling locations for each method overlap significantly in terms of their position along the growth increments of each shell. For the hand‐drilled samples to cover the regions of high δ^13^C but still to average out to the same ~1‰ value as the rest of the sequence, it would be necessary to suppose very localised areas of very high δ^13^C (which coincidentally aligned with the micromilled sampling locations) surrounded by areas of shell with δ^13^C values lower than 1‰ (which would also need to have been coincidentally missed by the higher resolution sampling). Given the extreme unlikelihood of this scenario, along with the aforementioned implausibility of DIC conditions that would result in such high values of δ^13^C, we do not consider this a feasible interpretation.

One other possible explanation for the differences in results between the micromilled and hand‐drilled samples could be the smaller sample size in the micromilled samples, as already discussed with reference to their higher failure rate. Particularly tiny samples can lead to greater errors, with uncertainty generally attributed to the enhanced influence of exchange with the reference gas during mass spectrometry. As anomalous results in our study appear to be present only in the δ^13^C results, mixing with the reference gas is only a plausible cause if the reference gas has a very similar δ^18^O value to the samples but a very different δ^13^C value. If such a scenario is correct, in order to cause high δ^13^C values the reference gas would have to show positive δ^13^C values. However, in this case the reference gas is slightly negative (~ −2‰) for both δ^13^C and δ^18^O. We can eliminate this as a likely explanation for the erroneous δ^13^C values. Moreover, the stable isotope facility at the BGS routinely runs small sample sizes successfully, down to 5 μg, with no loss of precision or anomalous values.

Instead, it is more likely that polyester embedding resin contaminated the aragonite powders during the micromilling sampling process. The possibility of resin contamination has been observed previously by the stable isotope facility at the BGS in results from embedded speleothems, although these issues were not further investigated at the time. On examination of the sampling locations for the high δ^13^C samples in Figure 3 there is no obvious difference visible between those and the other sampling tracks, and nothing unusual was noted about these samples during milling. However, the method of sample collection used during micromilling – the use of a scalpel to push the powder onto the edge of a razor blade – does mean that the blades of the scalpel and the razor blade both make contact with (and potentially scrape) the surface of the polyester resin. It therefore seems possible for trace amounts of the resin to be scraped into the samples without it being visually clear to the micromill operator. Given the relatively sporadic appearance of δ^13^C anomalies in the micromilled data, it appears that this type of contamination is unpredictable and difficult to avoid when using such manual sample retrieval methods. The continuous section of anomalously high δ^13^C in shell II_1 may also suggest that this kind of contamination can ‘linger’ in the mass spectrometer and affect subsequent samples.

We had hoped to be able to demonstrate more clearly that resin contamination was the cause of these high values, but unfortunately were unable to measure the resin alone on the Isoprime plus Multiprep due to the risk of instrument contamination. A resin sample measured on the EA as organic material produced an average δ^13^C value of −27.97‰ (σ = 0.15), but it does not represent the effect of resin contamination on samples measured by H_3_PO_4_ acid digestion on the Isoprime plus Multiprep. The EA completely combusts the organic material, while incomplete acid digestion of organic components on the Isoprime plus Multiprep could result in particular δ^13^C‐positive components of the resin being analysed rather than the compound as a whole. The previous work of Schöne et al^35^ helps to resolve this issue by the addition of known small quantities of resin to carbonate reference material for acid digestion, which better mimics the typical conditions required for sampling in palaeoclimatological studies.

Where there is concern about the possibility of resin contamination, a replacement embedding material could be used, but it would be necessary to gauge whether this replacement too would suffer the same problem if it were to contaminate the sample material. An alternative sample retrieval method, replacing the scalpel/razor blade approach, might also reduce the chance of contamination from the embedding material if it involved less physical contact with the embedding material. One alternative method was proposed by Sakai and Kodan^16^ in 2011, where milled powder is sucked up using a vacuum pump. While this method achieves a high (>90%) sample retrieval rate, it requires specialist equipment since the apparatus structure must be specifically modified to fit the particular type of reaction vial being used such that one design is not universally applicable between labs. Likely for these reasons, the technique has not been widely used.

As this study illustrates for *S. sachalinensis*, the sampling resolution gained by micromilling is not always necessary or may even be undesirable, producing problematically small samples and resolution at a high enough level to distract from seasonal patterns. In this case, rather than overcoming the issues of small sample size by changing powder collection methods, a simpler solution would be to avoid micromilling altogether. Hand drilling can provide a simpler alternative which eliminates the need for a large flat sampling surface and embedding materials, effectively bypassing the issue of resin contamination and powder loss during sample collection. For large and/or fast‐growing species such as *S. sachalinensis*, the reduced resolution provided by hand drilling is in our opinion outweighed by these benefits. The balance would change in favour of micromilling with very small specimens or slow‐growing species, or if specific research questions necessitate extremely high‐resolution sampling of faster‐growing species.

## 4 CONCLUSIONS

The question that prompted this study was whether a number of unusually high δ^13^C values in our micromilled dataset were an artefact of the approach taken to sample preparation and collection. By comparing the stable isotope values of micromilled samples with hand‐drilled samples from the same area on two halves of the same valve, we were able to show that the highest δ^13^C results only occurred in the micromilled samples. We are confident that they do not represent a true reflection of marine conditions.

We conclude that these high δ^13^C values are most likely related to contamination from the polyester resin in which the samples were embedded, which could have entered the powdered samples during retrieval post‐milling. During the period of analysis there were no instrumental issues, and the internal reproducibility of each analysis and standard results were all checked for consistency. Reference gases continued to show normal, negative δ^13^C values, which suggests that exchange with the reference gas in particularly small samples did not contribute to these high δ^13^C values. Although the resin we analysed on the EA mass spectrometer produced a strongly negative δ^13^C value, we suggest that the Isoprime mass spectrometer and Multiprep device used to analyse the original carbonate samples resulted in less complete breakdown of the resin (by phosphoric acid in the Isoprime plus Multiprep instrument as opposed to combustion in the EA) and so the measured sample was only contaminated by one or more δ^13^C‐positive component(s) of the resin. In order to further investigate the resin contamination issue, we recommend analysis of carbonate reference material with known (small) amounts of resin contamination to better understand how the resin affects δ^13^C values and how predictable this is; however, there is the possibility of contamination of the organic material within the mass spectrometer (hence not tried here). δ^18^O values appeared unaffected by contamination issues specifically relating to resin embedding.

A further outcome of this study is a direct comparison of micromilling versus hand‐drilling microsampling approaches in terms of spatial resolution, sample failure rates, ease of interpretation and accessibility. Whilst it can be tempting to opt for the increased resolution of micromilling wherever possible, in this case we conclude that the higher resolution gained from micromilling is not always advantageous. Practically speaking, hand drilling saves time and analytical costs. Our results also show that in a large and relatively fast‐growing species such as *S. sachalinensis*, hand‐drilled samples show clear and easily interpreted seasonal cycles with a lower sample failure rate than those obtained via micromilling. This will be of interest to researchers working on many other long‐lived and fast‐growing sentinel species commonly targeted for stable isotope palaeothermometry and marine conditions, for example the species noted by Mann et al.^43^ The hand‐drilling approach can effectively bypass the potential issue of contamination from embedding materials, and while some other methods have been discussed to mitigate this issue, avoiding embedding in the first instance is of course chief among these. Our results, however, suggest that in cases where sampling resolution must be particularly high, or where it is important to show extremes in δ^18^O as precisely as possible, then micromilling remains the best option. With increased knowledge of the interpretational consequences and relative sampling resolution of the method, researchers across the field of carbonate stable isotope thermometry should be better equipped to decide which is more appropriate for their material and specific research questions.

### PEER REVIEW

The peer review history for this article is available at https://publons.com/publon/10.1002/rcm.9318.

## Data Availability

The data that support the findings of this study are available from the corresponding author upon reasonable request.
